# Effect of self-assembly on fluorescence in magnetic multiphase flows and its application on the novel detection for COVID-19

**DOI:** 10.1063/5.0048123

**Published:** 2021-04-06

**Authors:** Xiang Li, Zhi-Qiang Dong, Peng Yu, Lian-Ping Wang, Xiao-Dong Niu, Hiroshi Yamaguchi, De-Cai Li

**Affiliations:** 1 Harbin Institute of Technology, Harbin 515063, China; 2 Guangdong Provincial Key Laboratory of Turbulence Research and Applications, Department of Mechanics and Aerospace Engineering, Southern University of Science and Technology, Shenzhen 518055, China; 3 Center for Complex Flows and Soft Matter Research, Southern University of Science and Technology, Shenzhen 518055, China; 4 College of Engineering, Shantou University, 243 Daxue Road, Shantou 515063, China; 5 Energy Conversion Research Center, Doshisha University, Kyoto 630–0321, Japan; 6 Department of Mechanical Engineering, Tsinghua University, Beijing 100084, China

## Abstract

In the present study, the magnetic field induced self-assembly processes of magnetic microparticles in an aqueous liquid (the pure magnetic fluid) and nonmagnetic microparticles in ferrofluid (the inverse magnetic fluid) are experimentally investigated. The microparticles are formed into chain-like microstructures in both the pure magnetic fluid and the inverse magnetic fluid by applying the external magnetic field. The fluorescence parameters of these self-assembled chain-like microstructures are measured and compared to those without the effect of magnetic field. It is found that the fluorescence in the pure magnetic fluid is weakened, because the scattering and illuminating areas are reduced in the microstructures. On the contrary, the fluorescence in the inverse magnetic fluid is enhanced, because more fluorescent nonmagnetic microparticles are enriched and become detectable under the effect of the magnetic dipole force and the magnetic levitational force, and their unnecessary scattering can be absorbed by the surrounding ferrofluid. The average enhancement of the fluorescence area ratio in the inverse magnetic fluid with 3 *μ*m nonmagnetic microparticles reaches 112.92%. The present work shows that the inverse magnetic fluid has advantages such as low cost, no scattering effect, stable fluorescence intensity, and relatively low magnetic resistance. In the end, a prototype design for the novel detection of coronavirus disease 2019 based on the magnetic field induced self-assembly in the inverse magnetic fluid is proposed, which could support the epidemic prevention and control.

## I. INTRODUCTION

Since the outbreak of atypical pneumonia caused by the severe acute respiratory syndrome coronavirus 2 (SARS-CoV-2), which was first reported in December 2019, the novel coronavirus disease (COVID-19) was spreading rapidly and showed a great threat to global public health.^1^ As of 18 February 2021, 109 594 835 COVID-19 cases have been confirmed, including 2 424 060 deaths. In the early report of the World Health Organization (WHO), the COVID-19 epidemic was associated with the Huanan Seafood Market in Wuhan, Hubei province, China, but the associated source in specific animal was not identified.^1^ Identification and diagnosis of the respiratory viral infections are important for stopping the epidemic, determining appropriate treatment, saving lives, and avoiding the unnecessary waste of medical resources. Therefore, accurate and sensitive detection of COVID-19 are needed. The typical clinical symptoms of COVID-19 cases include fever (temperature ≥37.3 °C, 94% cases), dry cough (79% cases), sputum (23% cases), fatigue (23% cases), and myalgia (15% cases).^2^ However, these clinical symptoms are common features in other virus-infected diseases, which are not unique for COVID-19. Moreover, some COVID-19 cases (asymptomatic carriers) do not have any symptoms. The ground-glass opacity (GGO) of the lungs observed by the chest computed tomography (CT) scan is the only way to clinically diagnose the COVID-19 cases before adopting the reverse transcription-polymerase chain reaction (RT-PCR).^3,4^ Currently, the virus nucleic acid RT-PCT becomes the standard diagnostic procedure to identify the COVID-19 cases. However, there are some drawbacks of these RT-PCR tests: (a) the testing process takes a long time (2–3 h); (b) the trained technician is needed because of the complicated operation; (c) the test samples are dangerous, which include the active virus; (d) the expensive equipment is required; and (e) false negatives and false positives on COVID-19 cases often occur (commonly need at least twice RT-PCR tests to identify). Another way to diagnose COVID-19 rapidly, simply, and accurately is the detection of antibodies of SARS-CoV-2^5,6^ because the immunoglobulin M (IgM) antibodies are acted as the front line of defense, which can be detected 3–7 days after the infection.^7^ Besides, the long-term immunity and immunological memory, i.e., the immunoglobulin G (IgG) antibodies, are detectable 10–15 days after the infection, which indicates that the infection has happened some time ago. Combination of the detection of IgM and IgG could draw the timelines of the virus infection history and provide support for the treatment of COVID-19 cases.

The IgM and IgG detection tests for human blood or oral mucosal transudate take 15–20 min, and both the ideal sensitivity and the ideal specificity of the detection tests could theoretically reach 100%. However, the real sensitivity and the real specificity of the detection tests on the clinical diagnosis of COVID-19 are only around 80% and 90%, respectively.^5,8,9^ The possible reasons include thw following: (a) the number of antibodies is lower than the detection limitation; (b) the time for the generation of these antibodies is different due to the difference of individual immune. When the detection limitation is high, an undetectable period with low concentrations of IgM and IgG exists because the IgM decreases and disappears 1–2 weeks after the infection while the IgG is too less to be detected at that time. Therefore, to increase the sensitivity and specificity, more antibodies should be detected and identified by improving the detection test. The typical solutions used in the detection tests are prepared by dispersing the gold nanoparticles or the ferroferric oxide (Fe_3_O_4_) microparticles in the phosphate buffer saline (PBS).^5,8,10^ The recombinant antigens are modified on the surface of those particles as the receptors to capture the targeted antibodies. During the detection process, the motion of each particle is uncontrollable due to various complicated forces acted on these particles, such as the hydrodynamic force, the gravitational force, the inertial force, the particle-particle interaction force, the Brownian force, etc. With an assumption that all the antibodies could be bonded by the receptors, some fluid mechanics phenomena may still damage the sensitivity of the detection tests. First, some particles in the testing channel may adhere on the wall or sediment at the bottom so that the antibodies carried on the surfaces of these particles could not be marked and detected. Second, the rest particles could not be well marked by the fluorescence indicator or be immobilized by the test line, because of the different velocities and random distribution of these particles.

To approach these problems, a technique of noncontact manipulation in micro/nanoscale, i.e., the magnetic field induced self-assembly in magnetic multiphase flows,^11–22^ is adopted in this work, which could manipulate the micro/nanoparticles by applying an external magnetic field. Self-assembly has been proved as a prospective, powerful, and controllable technique in building micro/nanostructures, which is widely used in microsensors, heat transfer devices, biomedicines, and targeted drugs.^13,14^ Generally, the magnetic field induced self-assembly can be cataloged into two types based on the driven forces: (a) self-assemble magnetic particles in nonmagnetic fluid^15^ (the pure magnetic fluid), and (b) self-assemble nonmagnetic particles in magnetic fluid (the inverse magnetic fluid).^16–18^ For the pure magnetic fluid,^19–22^ magnetic microparticles are dispersed in the base fluid (aqueous solution, saline, and oil), and the self-assembled chain-like microstructures of magnetic microparticles are formed under the effect of the magnet dipole force produced by the magnetic moment. For the inverse magnetic fluid,^23^ both magnetic nanoparticles and nonmagnetic microparticles are dispersed in the base fluid, and the self-assembled chain-like microstructures of nonmagnetic microparticles are formed under the effect of magnetic dipole force produced by the inverse magnetic moment acted on each microparticle, which resists the magnetization from the external magnetic field. The pioneering work of the self-assembled microstructures of magnetic fluid (ferrofluid) was studied by Bitter^20^ in 1932 and Elmore^21^ in 1938, in which the ferric oxide suspended in ethyl acetate and the ferroferric oxide dispersed in water with soap solution were applied, respectively. Then, Ganguly *et al.*^24^ experimentally investigated the magnetic field induced self-assembled structures of magnetic nanoparticles in a pulsatile flow. He *et al.*^25^ self-assembled the nonmagnetic polystyrene beads into photonic crystal by applying an external magnetic field to design the colloidal crystal-based optic device. Iwamoto *et al.*^26^ measured the thermal conductivity of self-assembled silver nanowires and demonstrated that 7% enhancement can be obtained by the ordered structures. Garcia-Torres *et al.*^27^ designed a tunable bidirectional nanoscale propeller by self-assembling a paramagnetic spherical nanoparticle and a ferromagnetic nanorod. The control technique and the theoretical approach of the propeller were then developed by Calero *et al.*^28^

The purpose of this work is to investigate the effect of the magnetic field induced self-assembly process on the fluorescence in magnetic multiphase flows and propose a novel detection for COVID-19 based on the magnetic field induced self-assembly technique. To compare the two self-assembly processes in the pure magnetic fluid and the inverse magnetic fluid and reveal their underlying mechanisms, a series of experiments for magnetic and nonmagnetic microparticles are performed. All the self-assembly experiments are observed under an optical microscope with a uniform magnetic field. The remainder of this article is organized as follows. In Sec. II, the preparation and characterization of materials are first introduced; the experimental setup to observe the magnetic field induced self-assembly and the corresponding fluorescence image in magnetic multiphase flows is then established by adopting a metallographic microscope with a 365 nm ultraviolet light. In Sec. III, the self-assembly processes and their effects on the fluorescence in magnetic multiphase flows are discussed, and the prototype design for the novel detection of the COVID-19 is proposed. Finally, the conclusions are drawn in Sec. IV.

## II. EXPERIMENTAL SECTION

### A. Preparation and characterization of materials

**FIG. 1.**
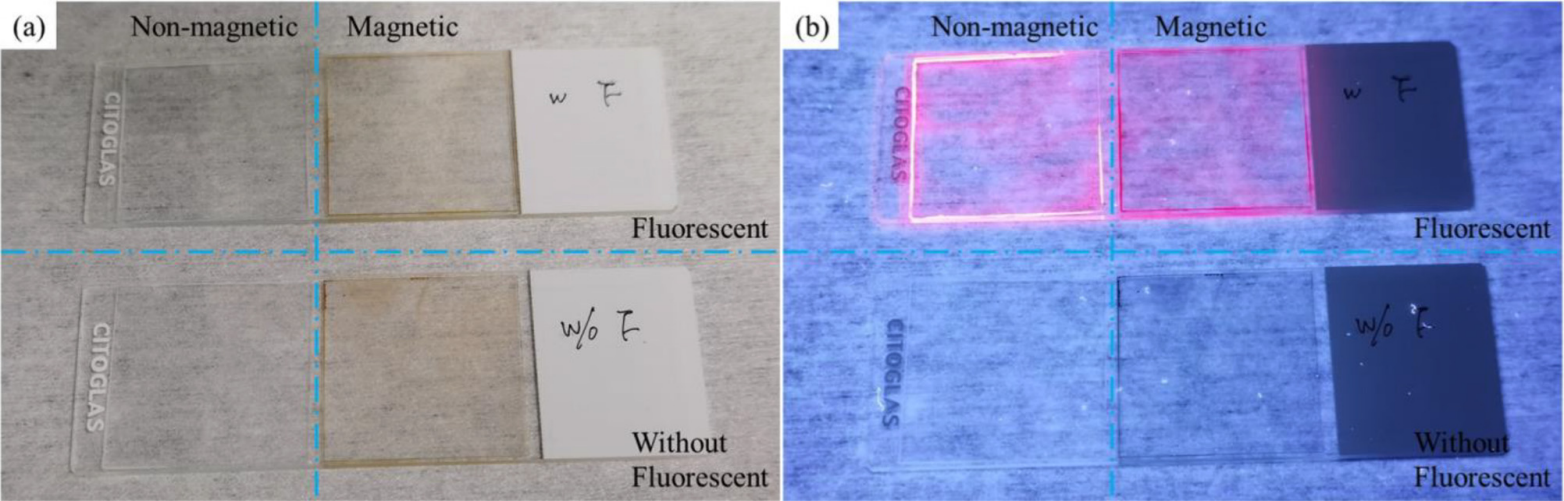
Comparison for the fluorescent responses of the 3 *μ*m nonmagnetic microparticles, the 3 *μ*m nonmagnetic fluorescent microparticles, the 3 *μ*m magnetic microparticles, and the 3 *μ*m magnetic fluorescent microparticles on glass slides: (a) white light; (b) 365 nm ultraviolet light.

The polystyrene microparticles are commonly used in the biomedicine and medical tests because of their unique advantages, such as good plasticity and durability, well-distributed particle diameter, and uniform shape with a good biocompatibility. Two types of magnetic multiphase fluids are used in this work: one is the pure magnetic fluid by dispersing the core-shell magnetic Fe_3_O_4_—polystyrene microparticles into deionized water (the common formula in the currently existing antibody detections) and the other is the inverse magnetic fluid by dispersing the nonmagnetic polystyrene microparticles into magnetic fluid (the suspension of magnetic Fe_3_O_4_ nanoparticles). The microparticles are synthesized by Bionano New Material Science (BNSM, Jiangsu, China), and their characterizations are shown in Table I. Based on the different surface modifications, these microparticles can be divided into two groups: one is the original microparticle; the other is the fluorescent microparticle which represents the antibodies for SARS-CoV-2 captured and carried on the particle after the preparing process of chemiluminescent immunoassay. Figure 1 shows the comparison for the fluorescent responses of the nonmagnetic microparticles, the nonmagnetic fluorescent microparticles, the magnetic microparticles, and the magnetic fluorescent microparticles on glass slides. Normally, the nonmagnetic microparticle solution on a slide is almost transparent, but the magnetic microparticle solution on a slide shows slightly yellowish. When applying the 365 nm ultraviolet light, the microparticle with a surface modification of europium emits a reddish orange light (615 ± 5 nm wavelength).

**TABLE I.** The characterization of microparticles.

No.	Type	Particle diameter (*μ*m)	Dispersion medium	Core material	Shell material	Surface modification	Excitation wavelength	Emission wavelength
1	Nonmagnetic	3, 5	Deionized water^a^	…	Polystyrene	-COOH	…	…
2	Nonmagnetic fluorescent	3, 5	Deionized water^a^	…	Polystyrene	-Europium	350–370 nm	615 ± 5 nm
3	Magnetic	3, 5	Deionized water^a^	Fe_3_O_4_	Polystyrene	-COOH	…	…
4	Magnetic fluorescent	3, 5	Deionized water^a^	Fe_3_O_4_	Polystyrene	-Europium	350–370 nm	615 ± 5 nm

^a^ 0.01% 2-Methyl-4-Isothiazolin-3-one (MIT, C_4_H_5_NOS) is added into de-ionized water.

**FIG. 2.**
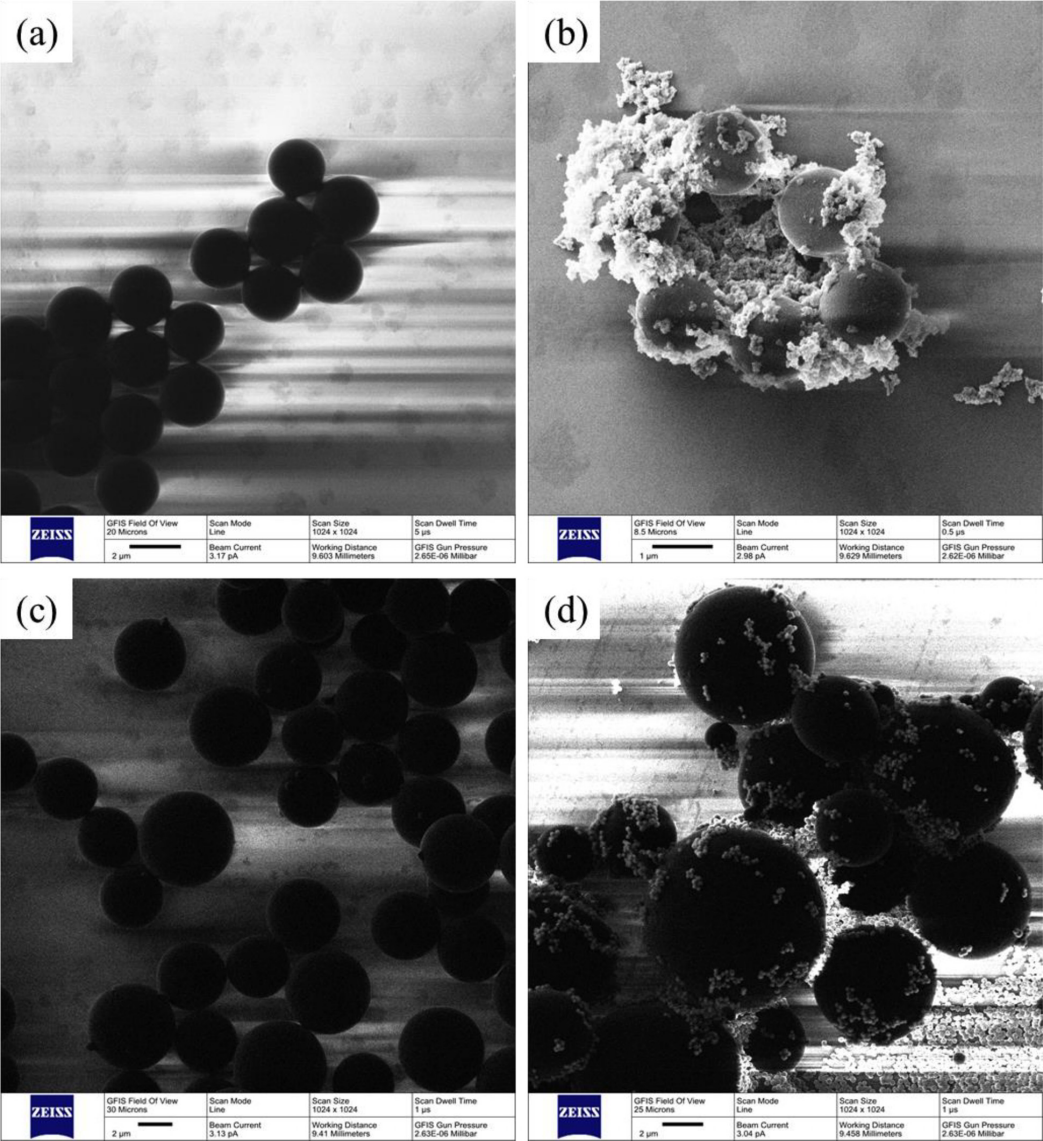
The surface topographies of the microparticles obtained by helium ion microscopy: (a) 3 *μ*m nonmagnetic microparticles, (b) 3 *μ*m nonmagnetic fluorescent microparticles, (c) 5 *μ*m magnetic microparticles, and (d) 5 *μ*m magnetic fluorescent microparticles.

The surface topographies of 3 *μ*m nonmagnetic microparticles, 3 *μ*m nonmagnetic fluorescent microparticles, 5 *μ*m magnetic microparticles, and 5 *μ*m magnetic fluorescent microparticles obtained by the Helium Ion Microscopy (HIM, Orion Nanofab, CARL ZEISS Germany) are shown in Figs. 2(a)–2(d), respectively. The microparticle diameters of the HIM samples are less than those suspended in fluid due to the freeze-drying process. Besides, the diameters of the nonmagnetic microparticles are more homogeneous than those of the magnetic microparticles with a polystyrene shell. Compared with the scanning electron microscope (SEM), the Helium beam at the typical acceleration voltage of 30 kV produces low energy distribution secondary electrons which only can escape from few nanometers of a sample, and thus the surface-sensitive image with the depth generated by the secondary electrons can be obtained. Besides, the metal spraying process is not necessary for the electrically insulating materials, because the positive charge on the test samples generating from the Helium beam can be easily compensated by a flood gun (a low voltage electron beam). Therefore, the original surface of these microparticles without the metal spraying process could be observed clearly.

In the present study, the magnetic Fe_3_O_4_ nanoparticles are produced by the coprecipitation method which includes two stages. In the first stage, the 40 ml iron (III) chloride solution (FeCl_3_, 1 mol) and 10 ml magnesium nitrate solution [Mg(NO_3_)_2_, 1 mol] are mixed, and then added into 350 ml sodium hydroxide solution (NaOH, 1 mol). After 5 min of heating and stirring, the red–brown precursors [FeOOH/Mg(OH)_2_] are gradually precipitated. In the second stage, the 400 ml iron (II) chloride solution (FeCl_2_, 0.3 mol) is added into the red–brown precursors under an intensive agitation with temperature of 70 °C, and then the mixed solution turns black immediately. The corresponding chemical reaction formulation reads

$$Fe^{2+}+2Fe^{3+}+8OH^{-}=Fe_{3}O_{4}+4H_{2}O.$$

To utterly react, the mixed solution in the second stage should be stirred over 25 min. After rinsing, filtering, and drying process, the magnetic ferroferric oxide (Fe_3_O_4_) nanoparticles are obtained. Further, the magnetic Fe_3_O_4_ nanoparticles are dispersed into the deionized water with surfactant.

### B. Experimental setup

**FIG. 3.**
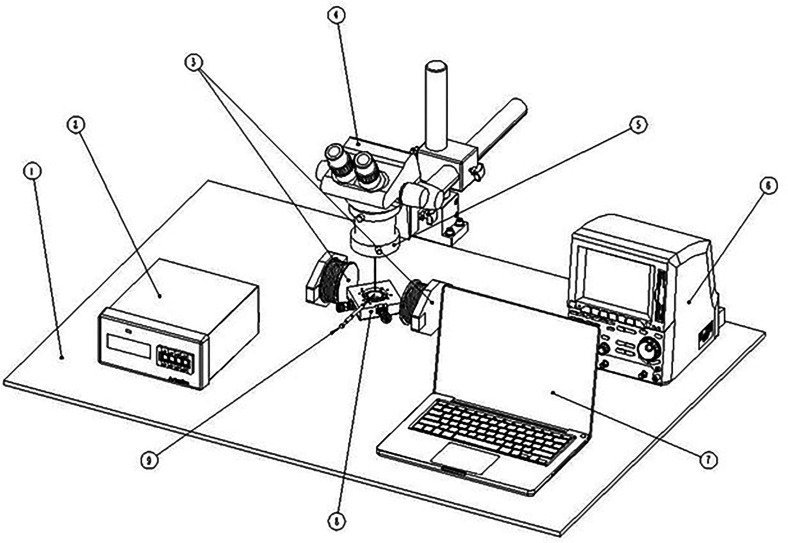
Experimental setup to observe the self-assembly process in the antibody detection under an external uniform magnetic field. (① The workbench, ② the monitor of Tesla meter, ③ a couple of electromagnets, ④ the electronic ocular of microscope (i.e., CCD camera), ⑤ the digital metallographic microscope, ⑥ the precision programable DC power supply, ⑦ the laptop, ⑧ the objective table with light source [365 nm ultraviolet light] and condenser lens, and ⑨ the probe of Tesla meter.).

**FIG. 4.**
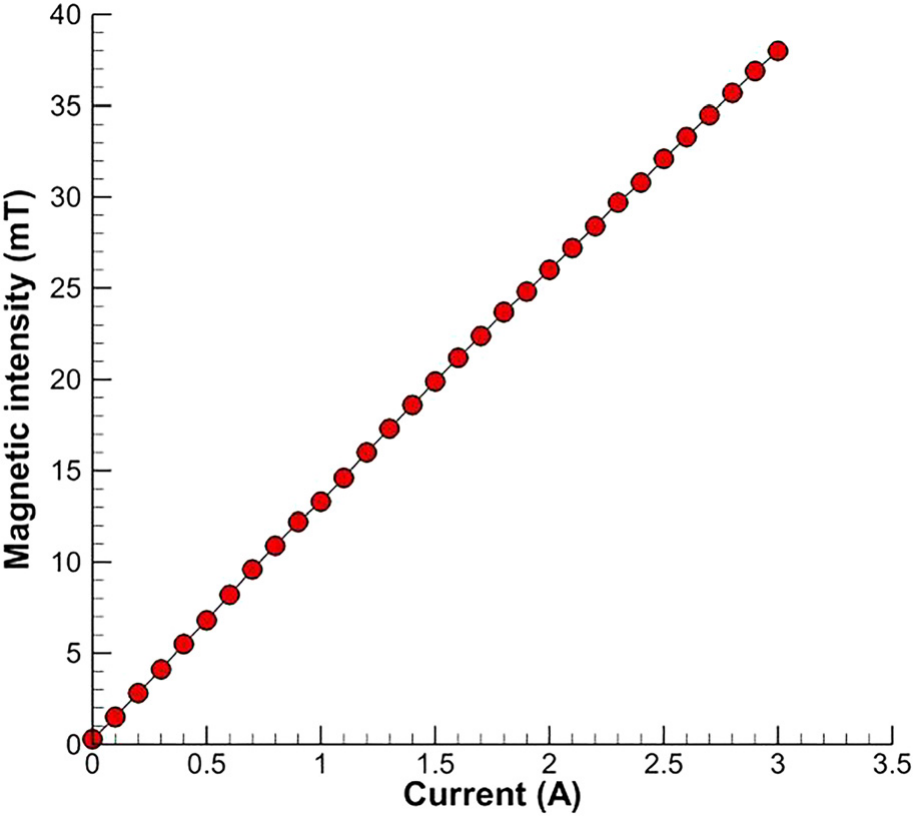
Variation of the magnetic intensity **H** with the current of DC power supply **A**.

The experimental setup to observe the self-assembly process in the antibody detection are shown in Fig. 3, which is composed of the workbench ①, the Tesla meter (monitor ② and its probe ⑨), the electromagnet ③, the electronic ocular of microscope (i.e., CCD camera) ④, the digital metallographic microscope ⑤, the precision programable DC power supply ⑥, the laptop ⑦, the objective table with light source (365 nm ultraviolet light) and condenser lens ⑧. The one-dimensional uniform magnetic field is produced by a couple of electromagnets (DC-MF, LANHAI INSTRUMENT, China), and the strength of magnetic field is controlled by the precision programable DC power supply (Model F2031, REF DEVICES, China) and measured and monitored by the Tesla meter (Model F1205, REF DEVICES, China). The unit of the Tesla meter is set as microTesla (mT) in the present experiments, which can be converted to Tesla (T) or Gauss (Gs) by the relation of 1 T = 1 × 10^3^ mT = 1 × 10^4^ Gs. Figure 4 shows the relation between the strength of the magnetic field (magnetic intensity **H**) and the current of DC power supply (**A**). The snapshots and videos of the magnetic field induced self-assembly in the antibody detection are magnified and observed by the digital metallographic microscope (XSP-63XDV, SHANGHAI OPYICAL, China) and recorded by the electronic ocular of the metallographic microscope (CCD camera, U3CCD06000KPA, SHANGHAI OPYICAL, China). The CCD camera used in the present work has 2748 × 2200 pixels.

### C. Experimental process

**FIG. 5.**
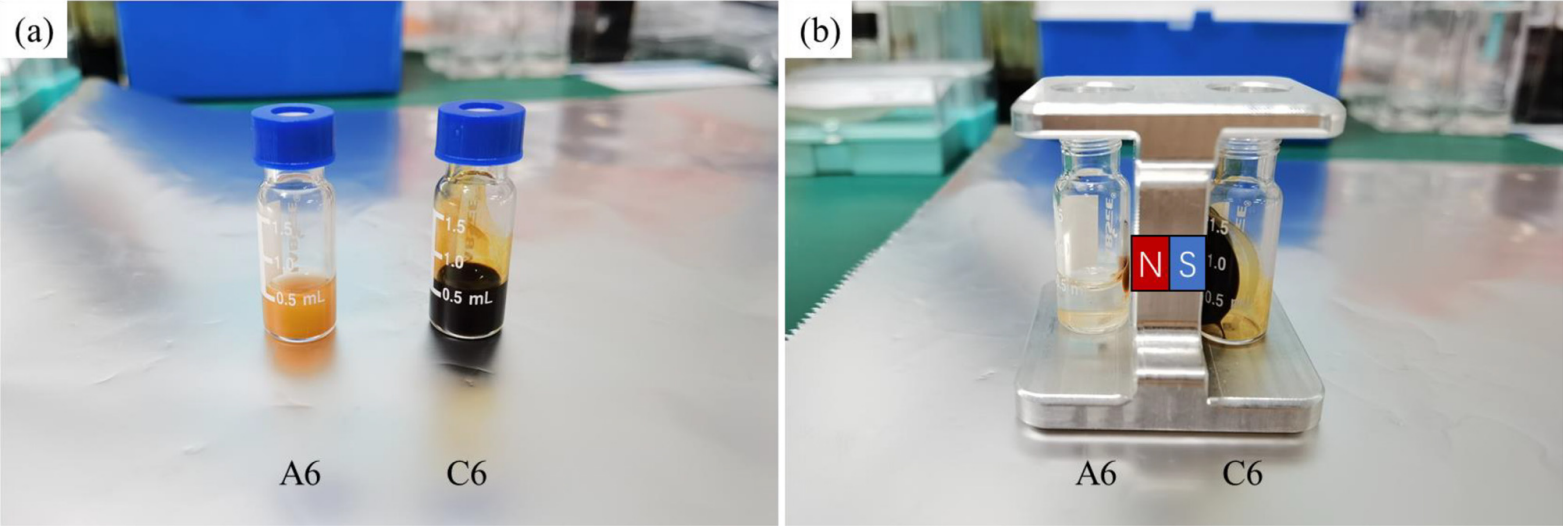
Comparison of the pure magnetic fluid and the inverse magnetic fluid used in the present work: (a) without magnet; (b) with magnet. The north and south poles of the magnet are indicated by the letters N and S.

Before the experiment, the test magnetic multiphase fluid comprising of the nonmagnetic polystyrene microparticles and the magnetic Fe_3_O_4_ nanoparticles should be prepared with two steps. First, a homogenous ferrofluid is prepared by dissolving the magnetic Fe_3_O_4_ nanoparticles in deionized water with 50% alcohol and 0.1% Tween-20 by using an ultrasonic homogenizer. The saturation magnetization of the ferrofluid is around 20 mT (200 Gs), and thus the external magnetic field should be set beyond 20 mT. Then, the nonmagnetic polystyrene microparticles are dispersed in the magnetic fluid by a stirring process or ultrasonic homogenizer. The concentrations of the test magnetic multiphase fluids are shown in Table II, with series A and B being the pure magnetic fluids and series C and D being the inverse magnetic fluids. The fluorescent microparticle ratio is defined by the ratio between the numbers of the fluorescent microparticles and the total microparticles, which presents the concentration of the targeted antibodies. In addition, the test magnetic multiphase fluid should be stirred immediately by a vibration mixer to avoid the sedimentation of nonmagnetic polystyrene microparticles before the experiment. Figure 5 compares the pure magnetic fluid (A06) and the inverse magnetic fluid (C06). The colors of the pure magnetic fluid and the inverse magnetic fluid are yellow and brownish black, respectively. When applying a magnetic field, the pure magnetic fluid turns transparent, because the magnetic microparticles are attracted and separated from the solvent. However, the inverse magnetic fluid remains stable, and all the solution is captured and maintained by the magnet. Besides, the cost of nonmagnetic microparticles is around 15 times cheaper than that of the magnetic microparticles. Although ferrofluid is more expensive than the pure magnetic fluid, the overall cost of the test solution based on the inverse magnetic fluid is around 10 times cheaper than that based on the pure magnetic fluid.

**TABLE II.** The concentrations of the test magnetic multiphase fluids.

No.	Total volume (*μ*l)	Magnetic microparticles	Magnetic fluorescent microparticles	Nonmagnetic microparticles	Nonmagnetic fluorescent microparticles	Ferrofluid	Fluorescent microparticle ratio (%)	Deionized water^a^ (μl)
A00	600	3 *μ*m, 50 mg	…	…	…	…	0	600
A02	600	3 *μ*m, 40 mg	3 *μ*m, 10 mg	…	…	…	20	600
A04	600	3 *μ*m, 30 mg	3 *μ*m, 20 mg	…	…	…	40	600
A06	600	3 *μ*m, 20 mg	3 *μ*m, 30 mg	…	…	…	60	600
A08	600	3 *μ*m, 10 mg	3 *μ*m, 50 mg	…	…	…	80	600
A10	600	…	3 *μ*m, 50 mg	…	…	…	100	600
B00	600	5 *μ*m, 50 mg	…	…	…	…	0	600
B02	600	5 *μ*m, 40 mg	5 *μ*m, 10 mg	…	…	…	20	600
B04	600	5 *μ*m, 30 mg	5 *μ*m, 20 mg	…	…	…	40	600
B06	600	5 *μ*m, 20 mg	5 *μ*m, 30 mg	…	…	…	60	600
B08	600	5 *μ*m, 10 mg	5 *μ*m, 50 mg	…	…	…	80	600
B10	600	…	5 *μ*m, 50 mg	…	…	…	100	600
C00	600	…	…	3 *μ*m, 50 mg	…	400 *μ*l	0	200
C02	600	…	…	3 *μ*m, 40 mg	3 *μ*m, 10 mg	400 *μ*l	20	200
C04	600	…	…	3 *μ*m, 30 mg	3 *μ*m, 20 mg	400 *μ*l	40	200
C06	600	…	…	3 *μ*m, 20 mg	3 *μ*m, 30 mg	400 *μ*l	60	200
C08	600	…	…	3 *μ*m, 10 mg	3 *μ*m, 50 mg	400 *μ*l	80	200
C10	600	…	…	…	3 *μ*m, 50 mg	400 *μ*l	100	200
D00	600	…	…	5 *μ*m, 50 mg	…	400 *μ*l	0	200
D02	600	…	…	5 *μ*m, 40 mg	5 *μ*m, 10 mg	400 *μ*l	20	200
D04	600	…	…	5 *μ*m, 30 mg	5 *μ*m, 20 mg	400 *μ*l	40	200
D06	600	…	…	5 *μ*m, 20 mg	5 *μ*m, 30 mg	400 *μ*l	60	200
D08	600	…	…	5 *μ*m, 10 mg	5 *μ*m, 50 mg	400 *μ*l	80	200
D10	600	…	…	…	5 *μ*m, 50 mg	400 *μ*l	100	200

^a^ 0.01% 2-Methyl-4-Isothiazolin-3-one (MIT, C_4_H_5_NOS) is added into deionized water.

The required strength of the external magnetic field is adjusted by the precision programable DC power supply cooperated with the Tesla meter. The current of DC power supply is set to 3.5 A, and the corresponding strength produced by the electromagnets is 32.1 mT. Then, the experimental chip, i.e., the microscopic glass slide with a coverslip (24 mm × 24 mm), is put into the objective table immediately to prevent the thin layer of the 5.5 *μ*l test magnetic multiphase fluid from evaporating on the chip. The distance between the slide and coverslip (the thin layer) is around 9.5 *μ*m. When applying the magnetic field, the magnetic field induced self-assembly process could be observed by the CCD camera and recorded by the laptop. Several observation areas of the experimental chip should be recorded and analyzed to obtain the average results. In this work, each test multiphase fluid is measured 2–3 times, and the effective data are obtained from over 10 observation areas. Self-assembly process starts immediately after applying an external magnetic field, and the stable chain-like microstructures are formed after 5 s. Thus, all measurements of self-assembled microstructures begin after 10 s to ensure reliable data. To quantitatively describe the fluorescence in the test magnetic multiphase fluids, the integrated fluorescence density, the fluorescence area, the mean fluorescence intensity, and the fluorescence area ratio are measured in this work.^29–32^ The integrated fluorescence density is obtained by integrating the gray value on each pixel. Then, the total pixels occupied by the fluorescence, which is called the fluorescence area, are recorded by using ImageJ software. The fluorescence area ratio is a dimensionless parameter, which is defined as the ratio between the fluorescence area and the overall pixels. The fluorescence area, together with the fluorescence area ratio, presents the amount of the fluorescence. Further, the mean fluorescence intensity can be obtained by calculating the ratio between the integrated fluorescence density and the fluorescence area.

## III. RESULTS AND DISCUSSION

In this section, the two types of the test magnetic multiphase fluids, i.e., the pure magnetic fluids (series A and B in Table II) and the inverse magnetic fluids (series C and D in Table II), are considered. The phenomena of magnetic field induced self-assembly in these two types of fluids are compared. Then, the quantitative comparisons of the fluorescence parameters, such as the integrated fluorescence density, the fluorescence area, the mean fluorescence intensity, and the fluorescence area ratio, are presented. Finally, the prototype design for the COVID-19 detection is proposed.

### A. The self-assembly phenomena and the fluorescence in the test magnetic multiphase fluids

The pure magnetic fluids, which are the commonly applied in the currently existing antibody detections, are experimentally tested under the microscope, and the self-assembled microstructures and the fluorescence in the pure magnetic fluid are observed in a glass chip. It should be pointed out that, in the existing antibody detections, the magnetic field is only used to achieve the magnetic separation (Fig. 5) in the preparation stage, and the magnetic field induced self-assembly is not involved in the later stage. However, for the purpose of comparison, an external uniform magnetic field is applied to self-assemble the magnetic microparticles in the pure magnetic fluid and its effect on the fluorescence is investigated in the present work.

**FIG. 6.**
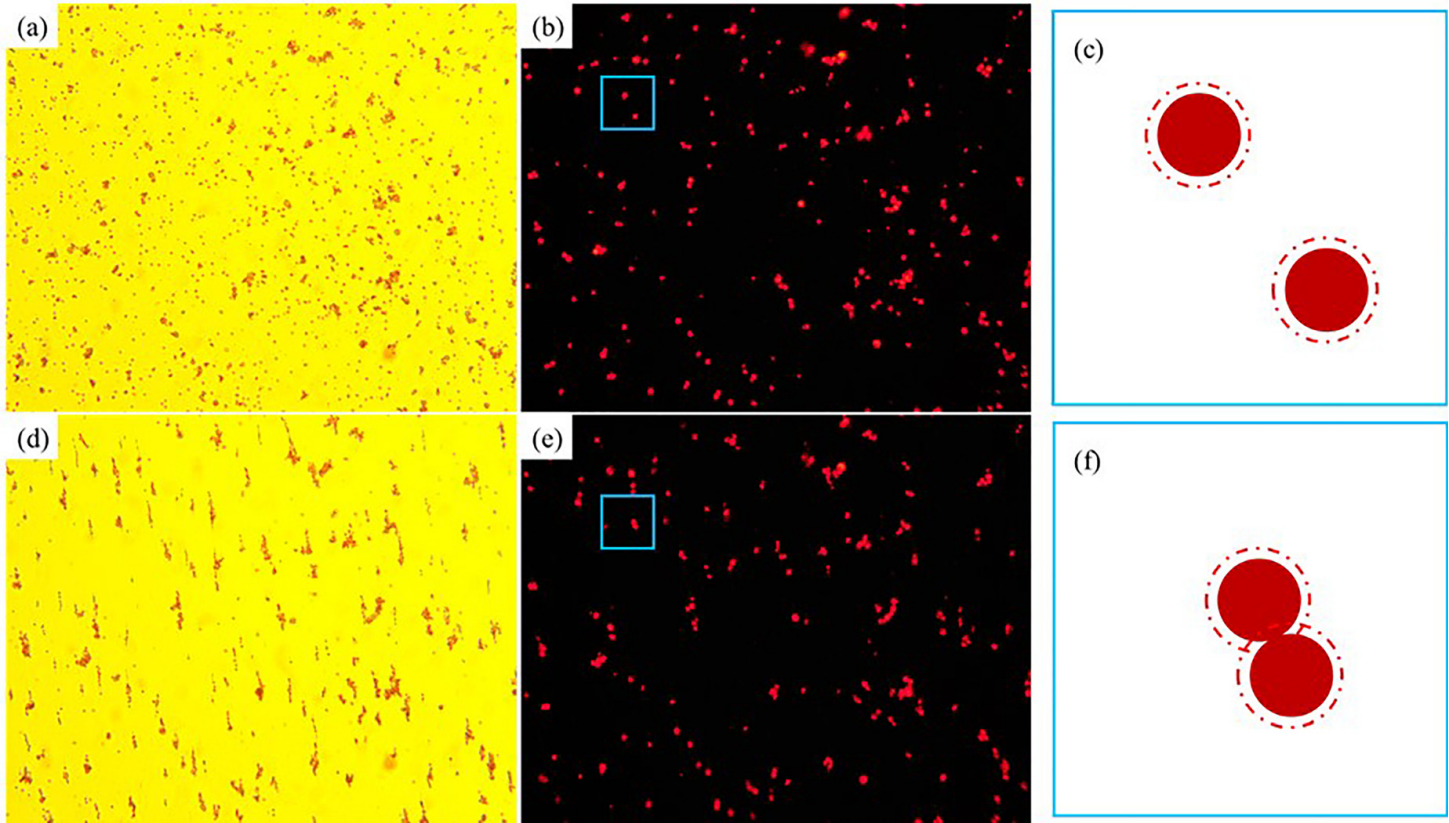
The magnetic field induced self-assembly process of 3 *μ*m magnetic microparticles in the A06 test magnetic multiphase fluid: (a) white light, without magnetic field; (b) 365 nm ultraviolet light, without magnetic field; (c) schematic diagram, without magnetic field; (d) white light, with magnetic field; (e) 365 nm ultraviolet light, with magnetic field; (f) schematic diagram, with magnetic field.

Figure 6 shows the magnetic field induced self-assembly process of 3 *μ*m magnetic microparticles in the A06 test magnetic multiphase fluid (the pure magnetic fluid). The distribution of magnetic microparticles and the fluorescent image under 365 nm ultraviolet light at the initial time are presented in Figs. 6(a) and 6(b), respectively. When an external uniform magnetic field is applied, the magnetic microparticles self-assemble into various chain-like microstructures, which are shown in Fig. 6(d). The corresponding fluorescent image is manifested in Fig. 6(e). The self-assembled microstructures include the double linear structure, the spindle-shaped structure, the branch-type structure, etc. The main reasons to form these structures include that the diameter of the magnetic microparticle could not be controlled well, the magnetic Fe_3_O_4_ core could not be perfectly ensured at the center of the microparticles, and the bulk of adhered microparticles shows a negative effect on the self-assembly process. A typical example of the self-assembly process of decentration microparticles is demonstrated in the blue rectangular region of Figs. 6(b) and 6(e), and the corresponding enlarged schematic diagrams are shown in Figs. 6(c) and 6(f), respectively. The centerline of these two self-assembled microparticles has an angle with respect to the vertical magnetic field direction because the upper and the lower magnetic Fe_3_O_4_ cores in Fig. 6(f) shift right and left from their centers, respectively. Besides, the fluorescent effect is crippled by the magnetic field induced self-assembly process. As shown in Fig. 5, the base fluid of the pure magnetic fluid is transparent. Thus, the fluorescence of the microparticles scatters and illuminates the surrounding liquid, which is sketched as the red dash circle in Fig. 6(c). When an external magnetic field is applied, these two microparticles self-assemble and contact, and thus the area illuminated by the fluorescence in the self-assembled chain-like microstructure is reduced. The fluorescence area ratios of Figs. 6(b) and 6(e) are 2.0843% and 1.8365%, respectively, which indicate a clear and detectable decrement of the fluorescent effect (around −11.89%) after applying the magnetic field.

**FIG. 7.**
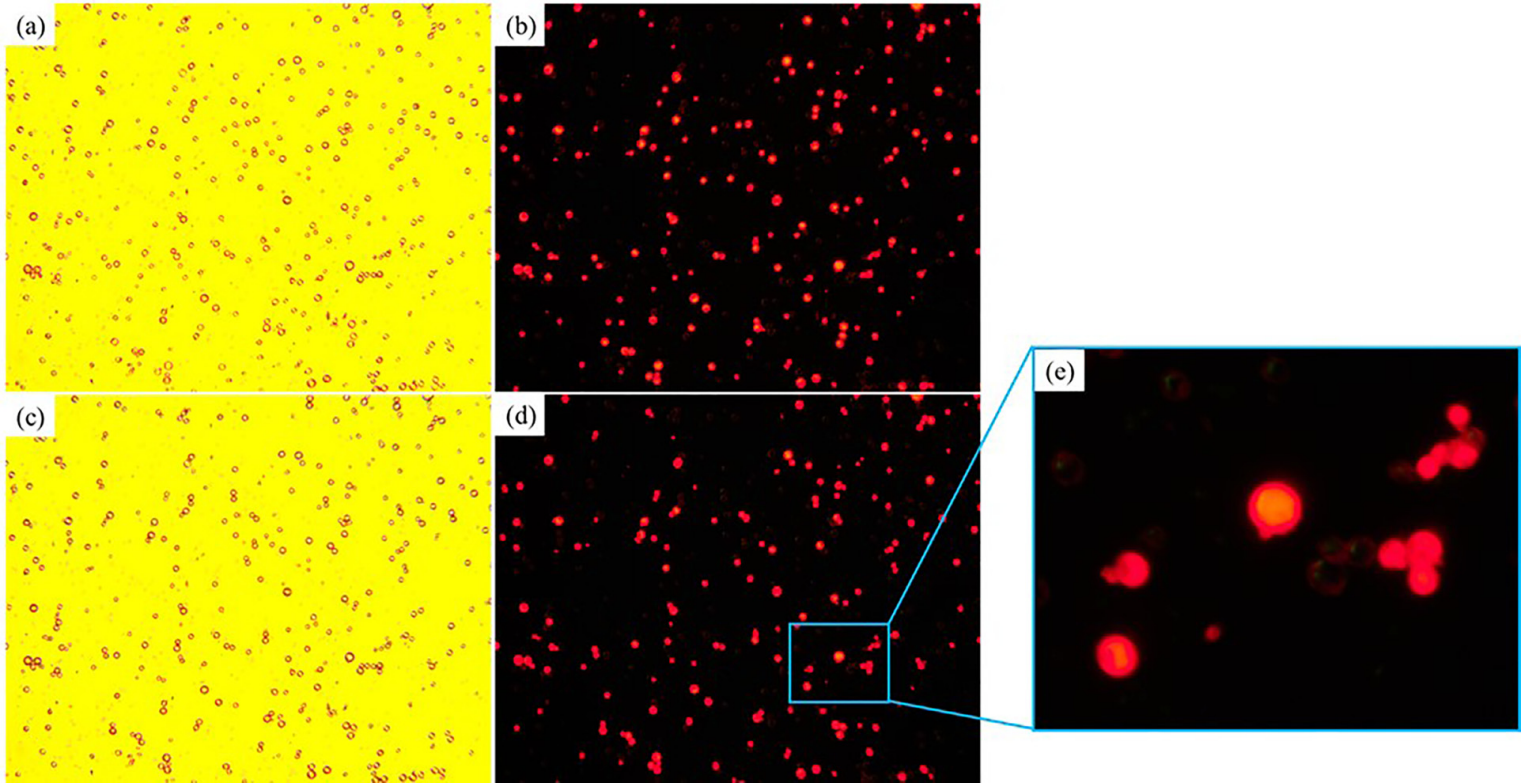
The magnetic field induced self-assembly process of 5 *μ*m magnetic microparticles in the B06 test magnetic multiphase fluid: (a) white light, without magnetic field; (b) 365 nm ultraviolet light, without magnetic field; (c) white light, with magnetic field; (d) 365 nm ultraviolet light, with magnetic field; (e) partial enlarged detail, with magnetic field.

The magnetic field induced self-assembled microstructures of 5 *μ*m magnetic microparticles in the B06 test magnetic multiphase fluid (the pure magnetic fluid) are shown in Fig. 7. The initial distribution of magnetic microparticles and the corresponding fluorescent image under 365 nm ultraviolet light are presented in Figs. 7(a) and 7(b), respectively. The nonhomogeneous diameter problem and the decentration of magnetic Fe_3_O_4_ core still exist. Only some weak motions of the magnetic microparticles can be observed under the external magnetic field, which is shown in Fig. 7(c). The main reason is that the magnetic force is relatively weak compared with the inertial force of the relatively big particle, and most self-assembled microstructures only consist of two magnetic microparticles. The corresponding fluorescent image is manifested in Fig. 7(d). The fluorescence area ratios of Figs. 7(b) and 7(d) are 4.5873% and 4.1333%, respectively. The reducing effect of fluorescence caused by the magnetic field induced self-assembly persists in this experiment, which is around −9.90%. Moreover, the magnetic microparticles without the surface modification of europium are illuminated by the neighboring fluorescent microparticles due to their scattering effect in the surrounding transparent liquid, which is demonstrated in the blue rectangular region of Fig. 7(d). The corresponding partial enlarged details are presented in Fig. 7(e). These two effects cause significant errors in the antibody detection. Therefore, the currently existing antibody detection methods are hard to obtain the quantitative results. However, the quantitative descriptions would be expected because they may offer the referable evidence to confirm the stage of the virus infection and retrospect the initial infection time.

**FIG. 8.**
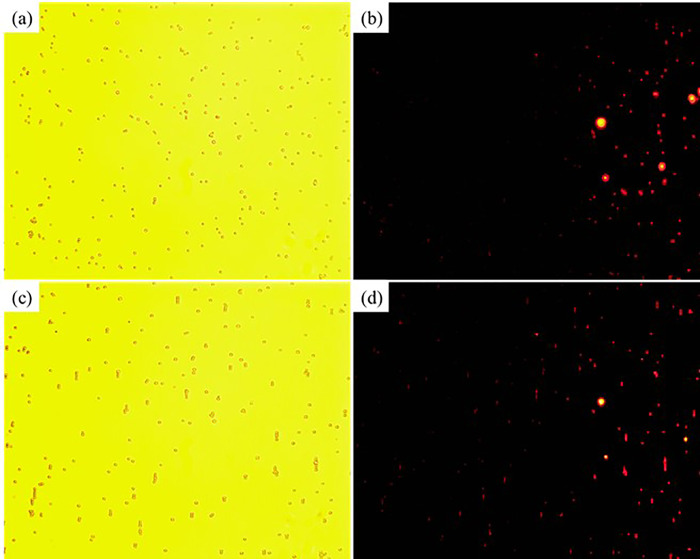
The magnetic field induced self-assembly process of 3 *μ*m nonmagnetic microparticles in the C06 test magnetic multiphase fluid: (a) white light, without magnetic field; (b) 365 nm ultraviolet light, without magnetic field; (c) schematic diagram, with magnetic field; (d) white light, with magnetic field.

The magnetic field induced self-assembly process of 3 *μ*m nonmagnetic microparticles in the C06 test magnetic multiphase fluid (the inverse magnetic fluid) is shown in Fig. 8. The initial distribution of the nonmagnetic microparticles and the corresponding fluorescence image are shown in Figs. 8(a) and 8(b), respectively. The nonmagnetic microparticles self-assemble into the chain-like microstructures after applying the external magnetic field, which are shown in Fig. 8(c). The corresponding fluorescent image is manifested in Fig. 8(d). The self-assembled chain-like microstructures also present single linear structure, double linear structure, spindle-shaped structure, and branch-type structure. However, the reasons for these various microstructures are different from those for the similar phenomena in Fig. 6(d). Unlike the magnetic microparticles, the diameters of the nonmagnetic polystyrene microparticles are homogeneous, and the decentration problem does not exist. The magnetic hysteresis in the nonmagnetic microparticles dominates the initial stage of self-assembly. The magnetization process of the nonmagnetic microparticles is not fully achieved, while the small particles are already driven by the magnetic dipole force. Besides, more nonmagnetic fluorescent microparticles could be detected in Fig. 8(d) than those in Fig. 8(b), because the magnetic dipole force and the magnetic levitational force drag and hold these microstructures on the focal plane of the microscope. Compared with the magnetic field induced self-assembly and the corresponding fluorescent experiments in Figs. 6 and 7, the reducing fluorescence effect caused by the magnetic field induced self-assembly is dispelled, because the scattering of the nonmagnetic fluorescent microparticles is absorbed by the surrounding black magnetic Fe_3_O_4_ nanoparticles. The fluorescence area ratios of Figs. 8(b) and 8(d) are 0.3593% and 0.5768%, respectively. The fluorescence area ratio increases 60.53% after the magnetic field induced self-assembly process.

**FIG. 9.**
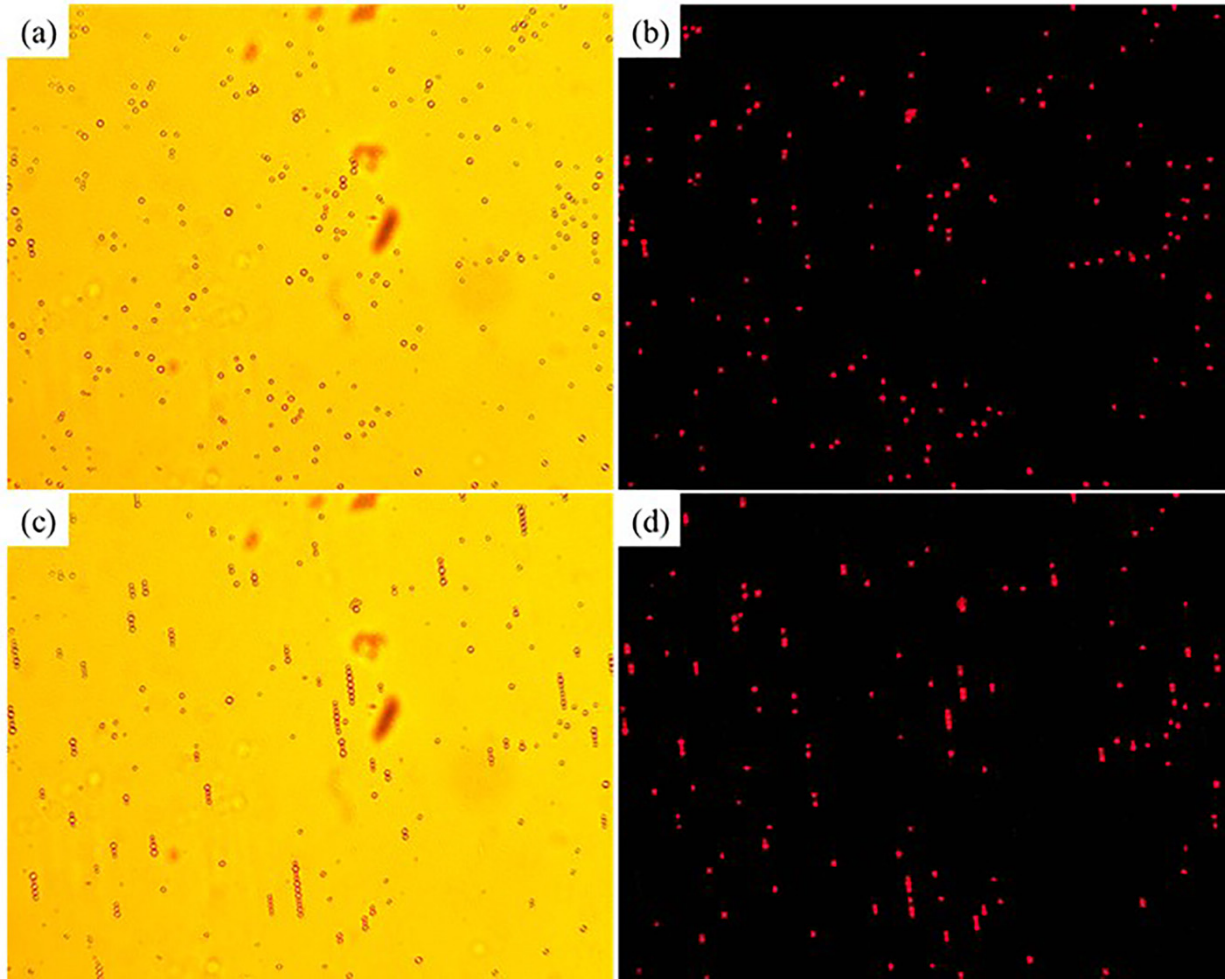
The magnetic field induced self-assembly process of 5 *μ*m magnetic microparticles in the D06 test magnetic multiphase fluid: (a) white light, without magnetic field; (b) 365 nm ultraviolet light, without magnetic field; (c) schematic diagram, with magnetic field; (d) white light, with magnetic field.

Figure 9 presents the magnetic field induced self-assembly process of 5 *μ*m magnetic microparticles in the D06 test magnetic multiphase fluid (the inverse magnetic fluid). The initial distribution of the nonmagnetic microparticles and the corresponding fluorescent image are shown in Figs. 9(a) and 9(b), respectively. Only the single linear microstructures are self-assembled by the external magnetic field, as shown in Fig. 9(c). The multiple shapes of the chain-like microstructures do not exist, because of the relatively bigger mass of 5 *μ*m magnetic microparticles, the boundary layer effects of the slide and coverslip, and the larger distance between two neighboring microparticles caused by the relative lower number density. With these effects, the microparticles move slowly and could be magnetized well at the initial stage of self-assembly. The fluorescence area ratios of Figs. 9(b) and 9(d) are 0.9919% and 1.2892%, respectively. Compared with the initial time, the fluorescence area ratio of the magnetic field induced self-assembly increases 29.97%.

### B. The fluorescence parameters of the test magnetic multiphase fluids

**FIG. 10.**
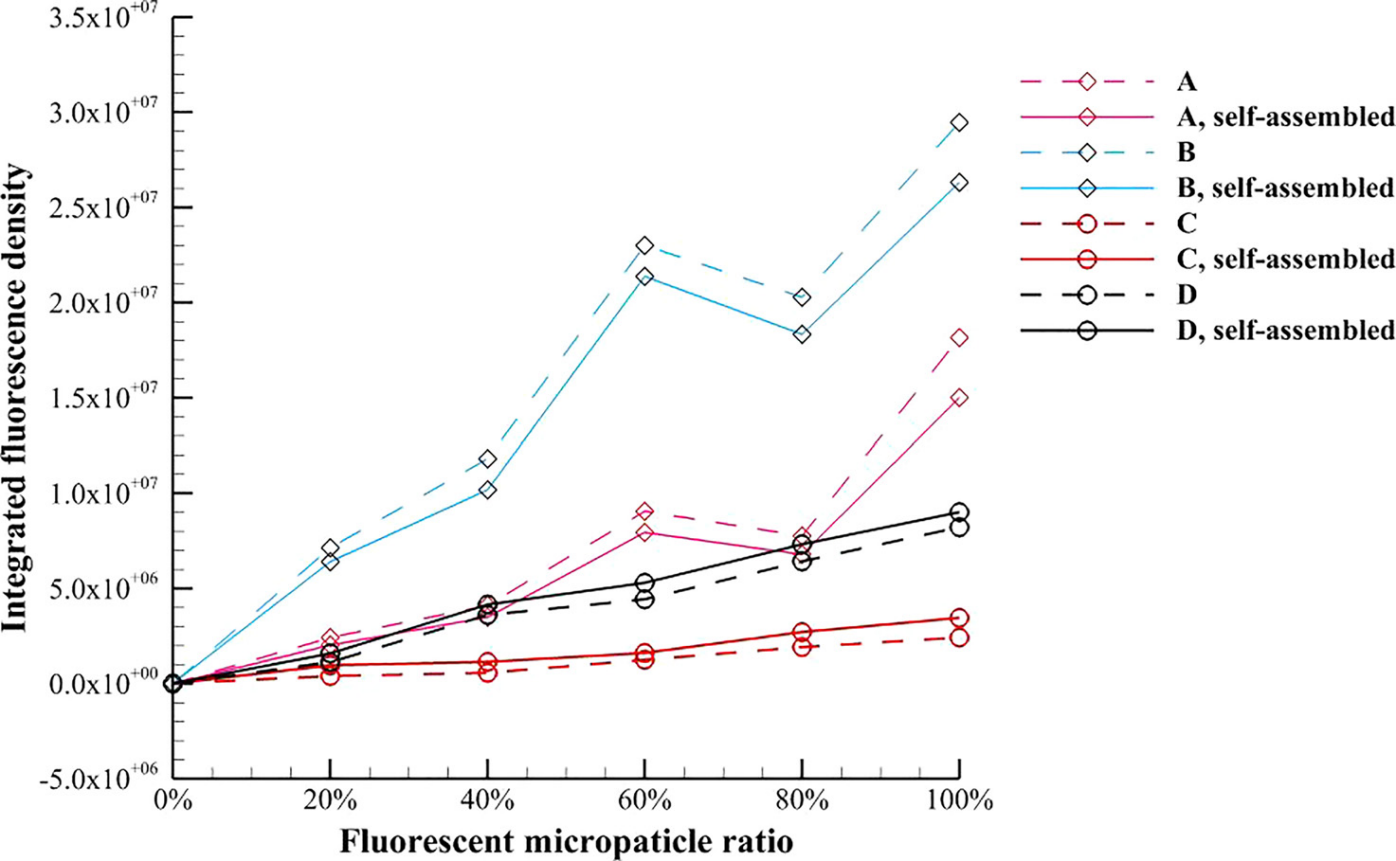
Variation of the average value of integrated fluorescence density of the test magnetic multiphase fluid with the fluorescent microparticle ratio.

In this subsection, the integrated fluorescence density, the fluorescence area, the mean fluorescence intensity, and the fluorescence area ratio of the pure magnetic fluid and the inverse magnetic fluid are experimentally quantified. Figure 10 shows the average value of integrated fluorescence density of the test magnetic multiphase fluid with different fluorescent microparticle ratios, which demonstrates that the integrated fluorescence density increases with the fluorescent microparticle ratio. For the pure magnetic fluid, a clear decrease in the integrated fluorescence density caused by the magnetic field induced self-assembly can be observed. When the fluorescent microparticle ratio is 60%, the peak values of the integrated fluorescent density appear for both 3 *μ*m and 5 *μ*m magnetic microparticles. The main reason is that the magnetic microparticles without surface modification of europium are illuminated by the neighboring magnetic fluorescent microparticles due to their scattering effect of the surrounding transparent liquid. The number density of the magnetic microparticles decreases when the magnetic fluorescent microparticle ratio is 80%, and thus fluorescent density is dominated by the magnetic fluorescent microparticles. This effect increases with the microparticle diameter.

Unlike the pure magnetic fluid, the integrated fluorescence density of the inverse magnetic fluid is monotonically increased due to the magnetic field induced self-assembly. The self-assembled chain-like microstructures gather the nonmagnetic fluorescent microparticles, which can enhance the fluorescence area. Moreover, the integrated fluorescence density of the inverse magnetic fluid is lower than that of the pure magnetic fluid. The main reason is that the fluorescence of the magnetic fluorescence microparticles scatters and illuminates in the surrounding transparent liquid, but the fluorescence of the nonmagnetic fluorescence microparticles is absorbed by the surrounding black Fe_3_O_4_ nanoparticles.

**FIG. 11.**
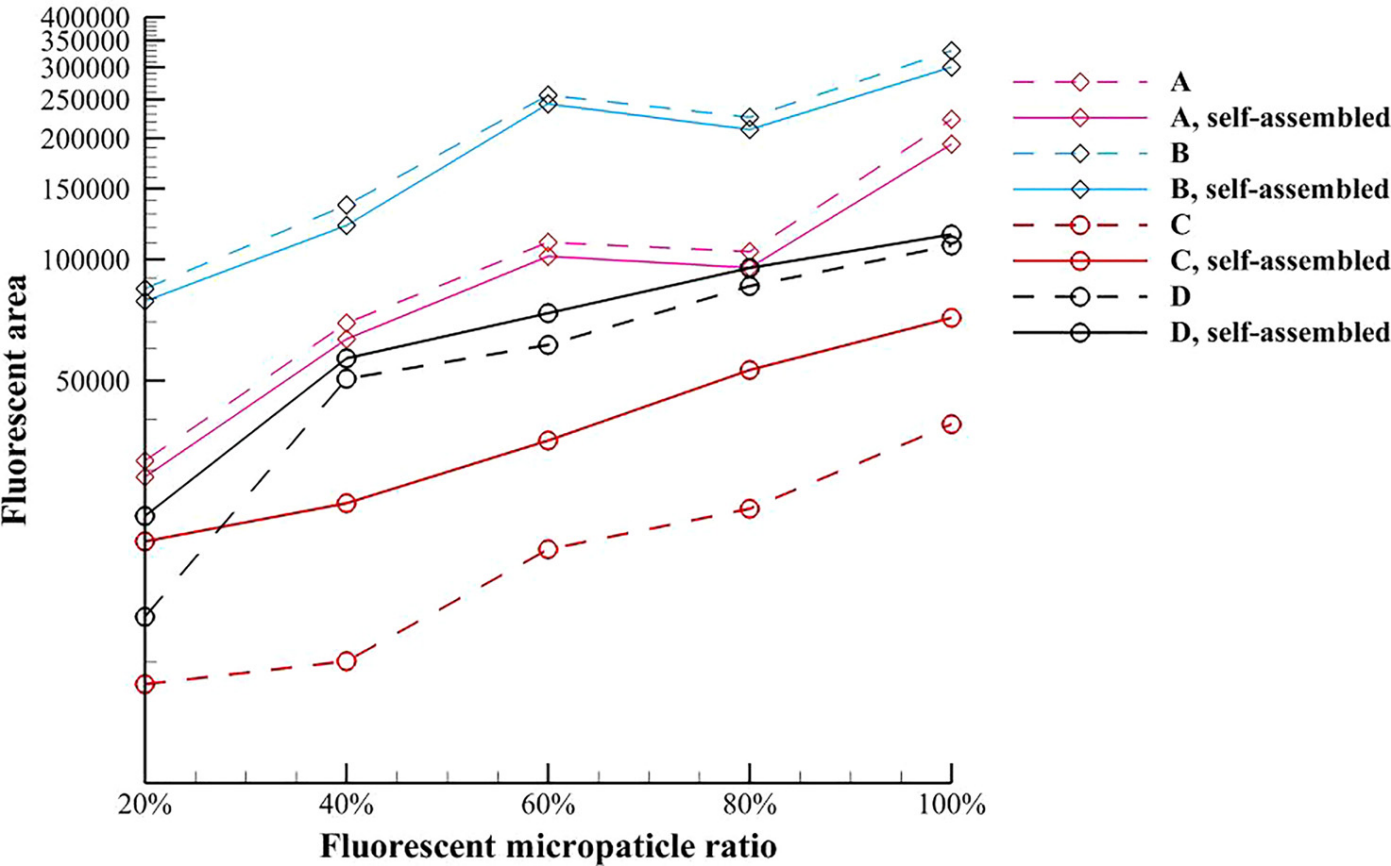
Variation of the average value of fluorescence area of the test magnetic multiphase fluid with the fluorescent microparticle ratio.

**FIG. 12.**
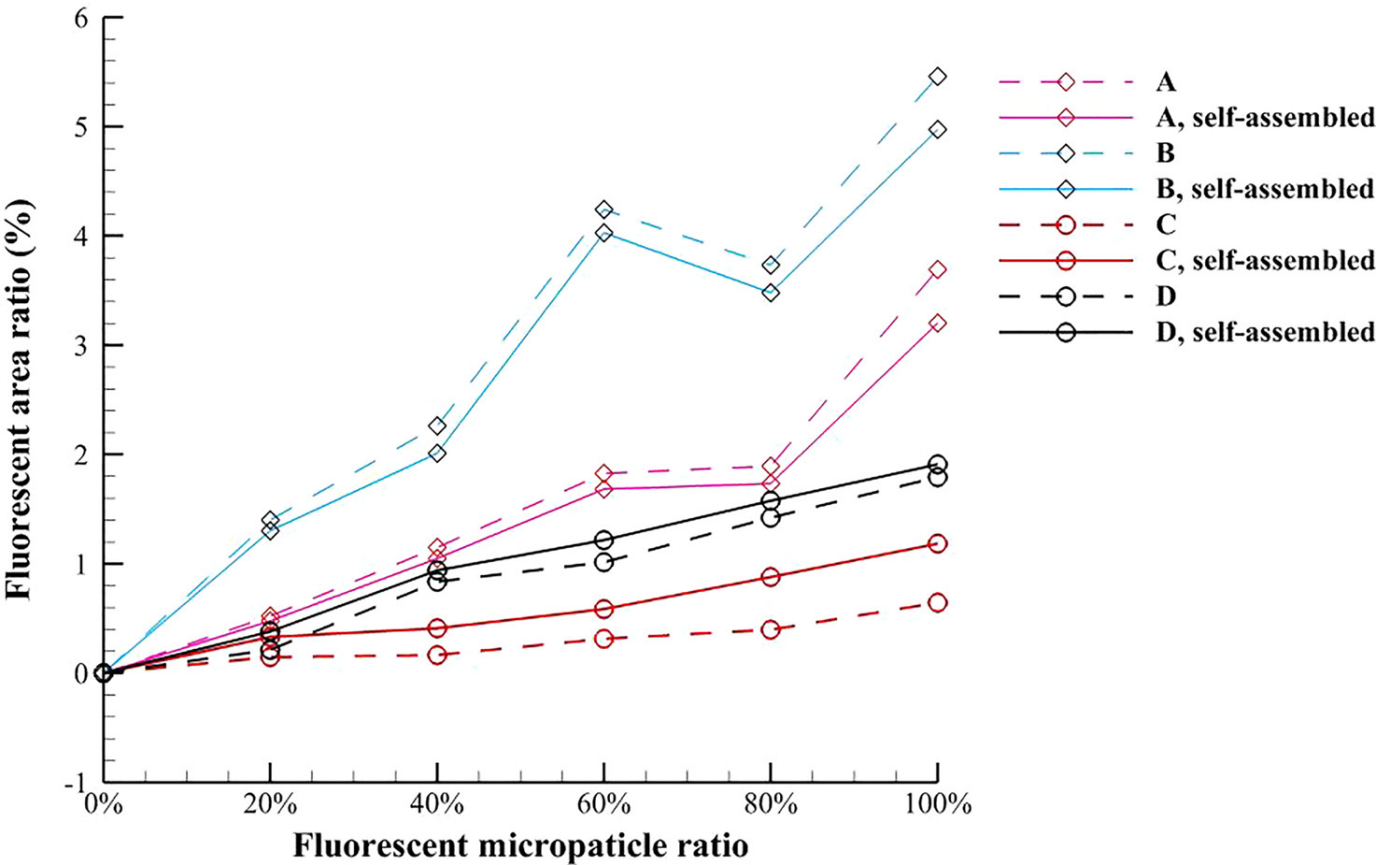
Variation of the average value of fluorescence area ratio of the test magnetic multiphase fluid with the fluorescent microparticle ratio.

The average values of fluorescence area and fluorescence area ratio of the test magnetic multiphase fluids are presented in Figs. 11 and 12, respectively. For the pure magnetic fluid, the decrease in fluorescence area caused by the magnetic field induced self-assembly results from the reduction of the fluorescent scattering area described in Figs. 6(c) and 6(f). Figure 12 denotes that the decreasing magnitude of the fluorescence area ratio increases with the diameter of magnetic microparticle. For the inverse magnetic fluid, the fluorescence area is monotonically increased because of the self-assembled chain-like microstructures. When the diameter of nonmagnetic microparticle is relatively smaller, this enhancement effect is even more pronounced. Besides, it is found that the enhancement magnitude in Fig. 12 increases with the fluorescent microparticle ratio when the smaller nonmagnetic microparticle is used. The average magnitudes of the fluorescent enhancement of 3 *μ*m and 5 *μ*m nonmagnetic microparticles are 112.92% and 25.57%, respectively.

**FIG. 13.**
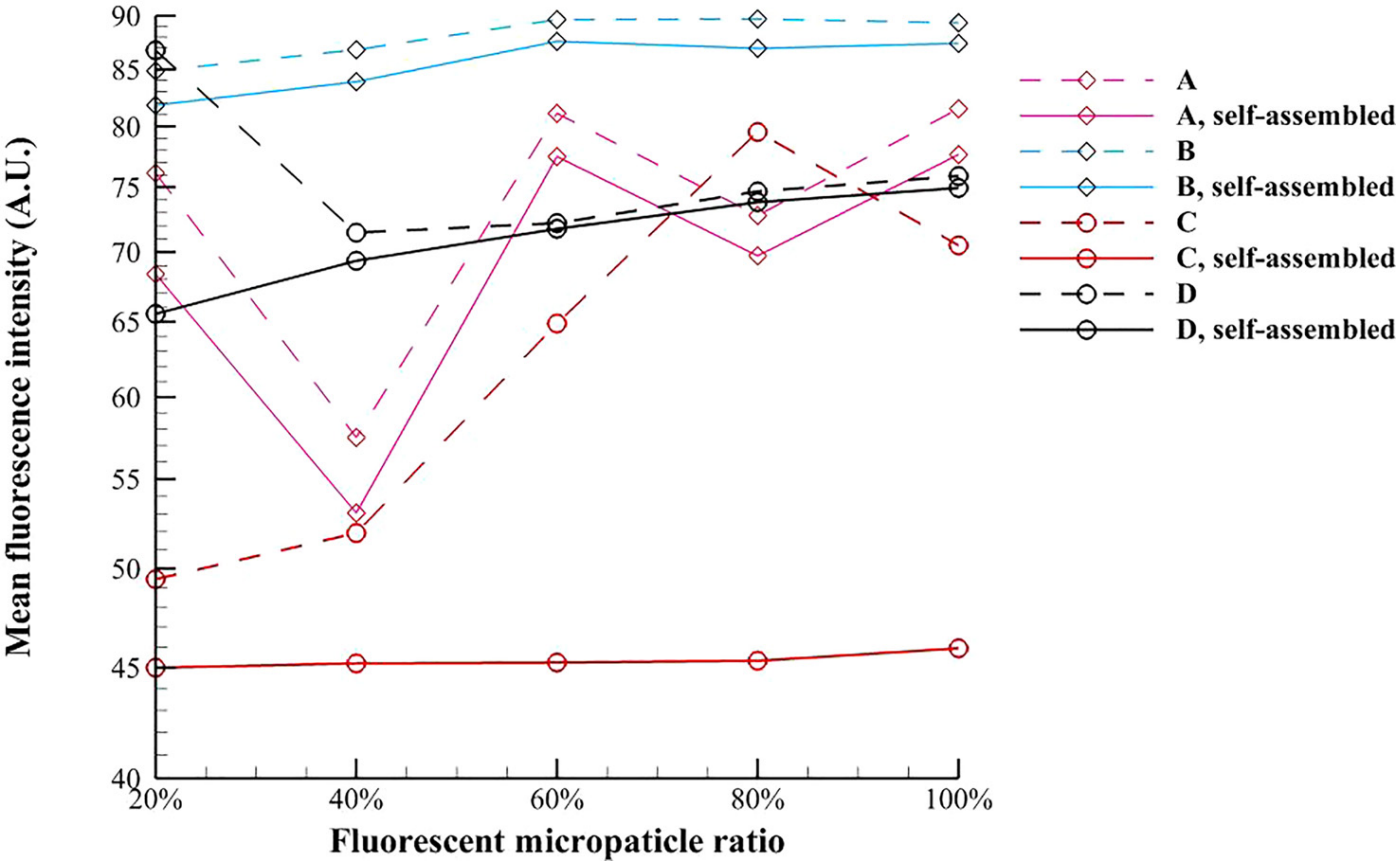
Variation of the average value of mean fluorescence intensity of the test magnetic multiphase fluid with the fluorescent microparticle ratio.

It should be noticed that the mean fluorescence intensity in the present work is not equal to the optical density, and thus the unit of the mean fluorescence intensity is arbitrary. Figure 13 presents the variation of the average value of mean fluorescence intensity of the test magnetic multiphase fluid with the different fluorescent microparticle ratios. It is found that the mean fluorescence intensities of both the magnetic and nonmagnetic microparticles under the external magnetic field are lower than the corresponding values at the initial time. For the pure magnetic fluid, the mean fluorescence intensity of self-assembled chain-like microstructures is more stable when the magnetic microparticle is relatively larger. On the contrary, the mean fluorescence intensity of self-assembled chain-like microstructures in the inverse magnetic fluid is more stable when the nonmagnetic microparticle is relatively smaller, and the average fluorescence intensity is around 45.3418.

### C. A prototype design for the novel detection of COVID-19

To quantitatively detect and assay the antibody, it requires a stable fluorescence intensity which should not change with the fluorescent microparticle ratio. The comprehensive analysis of the above experimental results indicates that the inverse magnetic fluid possesses some advantages in the antibody detection. First, the cost of nonmagnetic microparticles is cheaper than that of the magnetic microparticles, and the size of the nonmagnetic microparticles is easily controlled. Second, the magnetic Fe_3_O_4_ nanoparticles of the inverse magnetic fluid can absorb the redundant light scattered from the nonmagnetic fluorescent microparticles. Third, the magnetic field induced self-assembly process in the inverse magnetic fluid can enhance the fluorescent effect, and the fluorescence intensity of relatively smaller nonmagnetic microparticles is more stable.

Moreover, the magnetic field intensity at the center of nonmagnetic microparticle in the inverse magnetic fluid is higher than that of magnetic microparticles in the pure magnetic fluid. The magnetic potential equation for the circular cylinder under a uniform magnetic field can be written as^15^

$$\frac{\partial }{\partial r}\left(r\frac{\partial \phi }{\partial r}\right)+\frac{1}{r}\frac{\partial^{2}\phi }{\partial \theta^{2}}=0,$$ (1)

where *ϕ* is the magnetic potential, *r* and *θ* are the radial and angular coordinates, respectively. By applying the method of separation of variable, the analytical solution of the magnetic field intensity inside the circular cylinder can be obtained as^33,34^

$$H=-\nabla \psi =\frac{2\mu_{f}}{\mu_{p}+\mu_{f}}H_{0}\;\text{sin}\;\theta e_{r}+\frac{2\mu_{f}}{\mu_{p}+\mu_{f}}H_{0}\;\text{cos}\;\theta e_{\theta }.$$ (2)

For the pure magnetic fluid, the magnetic permeability of the microparticles (*μ_p_*) is higher than the magnetic permeability of the base fluid (*μ_f_*). The magnetic resistance of the base fluid impedes the conduction of the magnetic field. On the contrary, the magnetic permeability of the base fluid is much higher than that of nonmagnetic microparticle in the inverse magnetic fluid, and the magnetic resistance of the base fluid is very low since the ferrofluids are superparamagnetic.^35,36^ The exact value of magnetic permeability is hard to measure in the present study. Generally, the relative magnetic permeability of the magnetic material ranges from 1000 to 5000, and the relative magnetic permeability of the nonmagnetic material is close to 1. The magnetic resistance of the inverse magnetic fluid is around the order of 10^3^ smaller than that of the pure magnetic fluid. Therefore, the magnetic field intensity at the center of nonmagnetic microparticle in the ferrofluid is relatively higher than that at the center of magnetic microparticle in the aqueous liquid. The magnetic intensity of the external magnetic field required in the self-assembly process of the inverse magnetic fluid is lower than that of the pure magnetic fluid.

**FIG. 14.**
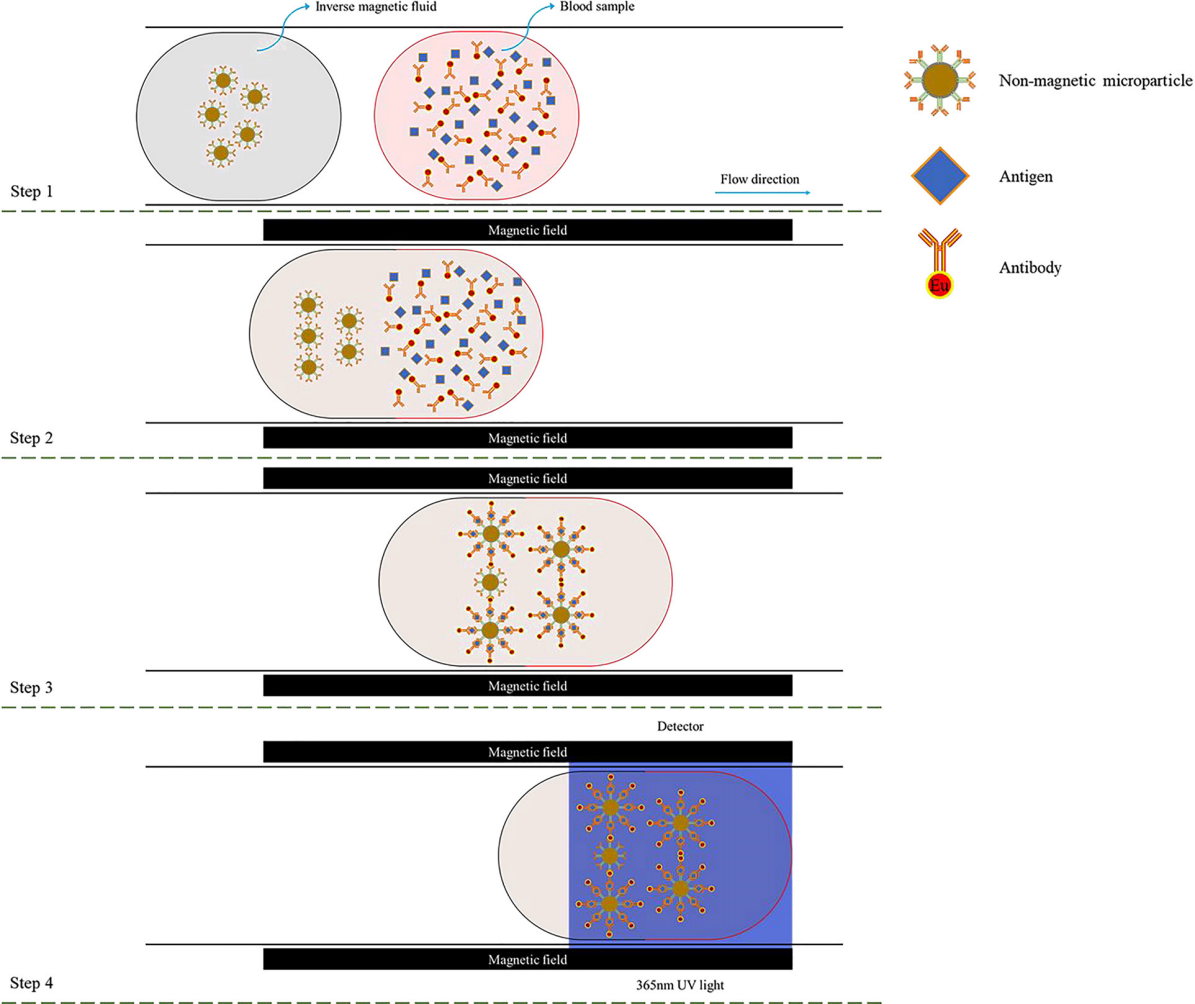
The detection process for COVID-19 based on the magnetic field induced self-assembly.

According to these advantages mentioned above, the detection process for COVID-19 based on the magnetic field induced self-assembly in the inverse magnetic fluids is presented in Fig. 14.^37,38^ Before the detection, the inverse magnetic fluid composing of the ferrofluid and nonmagnetic microparticles with the surface modification of second antibodies should be prepared, and the blood sample should be processed by marking the targeted antibodies with fluorescence labels (e.g., europium) and adding the specific antigens. In the first step, the inverse magnetic fluid and the test blood sample are injected into a microchannel. Then, a couple of electromagnets to generate an external uniform magnetic field are set at the microchannel, and the magnetic field intensity of them can be controlled by a DC power supply. The self-assembly process of nonmagnetic microparticles in the inverse magnetic fluid starts immediately after applying the external uniform magnetic field. Further, the inverse magnetic fluid and test blood sample blend together. The antigens act as a bridge between the targeted antibodies and the second antibodies of the nonmagnetic microparticles, and thus the targeted antibodies could be captured by and linked on the nonmagnetic microparticles. Finally, a 365 nm ultraviolet light is applied to the detection area. The fluorescence label is excited by the ultraviolet light and emits a reddish orange light (615 ± 5 nm wavelength) that can be recorded and analyzed by the detector. If any fluorescence area can be detected, the blood sample is positive. Otherwise, the blood sample is negative.

The present fluorescence enhancement in the inverse magnetic fluid by applying the external magnetic field is validated in the quiescent solution. In fact, most existing methods of the antibody detection require a motionless flow field or slow flow field, which is restricted by the frame rate of the camera (i.e., the frames per second, fps). To maintain the cost, the fps of the camera is not high. Therefore, to capture the clear pictures of fluorescence, the flow velocity of the test sample during the detection process should be slow. Actually, the self-assembled microstructures can be also formed in Poiseuille flow as demonstrated by Zhang *et al.*^39^ The patterns of the chain-like microstructures are determined by the flow speed and the strength of external magnetic field. Thus, the present detection method can also be applied to other flow conditions with slow velocity.

Table III presents the fluorescent microparticle ratios measured by the existing antibody detection method and the present antibody detection method, which are compared with the real fluorescent microparticle ratio. During the measurement, the fluorescence area ratio is first obtained by the experimental method mentioned above, and the fluorescent microparticle ratio is then obtained from Fig. 12. However, the relation between the fluorescence area ratio and the fluorescent microparticle ratio is not monotonical in the pure magnetic fluid due to the scattering and illuminating effect, and one fluorescence area ratio may correspond to two or three different fluorescent microparticle ratios. Thus, the fluorescent microparticle ratio ranging from 0.5 to 0.9 cannot be precisely measured in the existing detection method. It is found that the relative measurement error of the present method is less than that of the existing method and its absolute value decreases with an increase in the real fluorescent microparticle ratio, because the fluorescence area ratio monotonically increases with the fluorescent microparticle ratio. Compared with the existing antibody detection method, i.e., using the pure magnetic fluid without magnetic field induced self-assembly process, the proposed novel antibody detection method could provide the quantitative results of the antibodies, which may help to identify the stage of the virus infection and retrospect the initial infection time.

**TABLE III.** The fluorescent microparticle ratio measured by the antibody detection methods.

No.	True value	Existing method	Error (%)	Present method	Error (%)
1	0.1	0.0606	−39.4	0.0913	−8.7
2	0.3	0.3561	18.7	0.2836	−5.47
3	0.5	0.5424/0.7820	8.48/56.4	0.4809	−3.82
4	0.7	0.5501/0.7923	−21.4/13.19	0.7203	2.90
5	0.9	0.9691	7.67	0.9128	1.42

## IV. CONCLUSION

Magnetic multiphase flows widely occur in microfluidics, drug delivery, and targeted drugs. The magnetic field induced self-assembly in the magnetic multiphase fluid can be regarded as one of the efficient ways to construct the specific structure in microscale and nanoscale. To investigate the effect of magnetic field induced self-assembly on fluorescence in magnetic multiphase flow, the experimental setup based on the optical microscope is built, and the relative materials are prepared. The surface topographies of the original microparticles and the fluorescent microparticles are observed by using HIM, and the corresponding fluorescence responses are compared under a 365 nm ultraviolet light. Then, a series of experiments by using the magnetic microparticles in aqueous liquid and the nonmagnetic microparticles in ferrofluid are carried out. The self-assembled chain-like microstructures in two kinds of magnetic multiphase flows, i.e., the pure magnetic fluids and the inverse magnetic fluids, are experimentally investigated. The fluorescence parameters of these microstructures are measured and compared with that without the effect of magnetic field, such as the integrated fluorescence density, the fluorescence area, the mean fluorescence intensity, and the fluorescence area ratio. It is found that the fluorescence in the pure magnetic fluids is weakened by the self-assembly process, because the scattering and illuminating areas are reduced in the self-assembled chain-like microstructures. However, the fluorescence is enhanced by the self-assembled chain-like microstructures under the effect of the magnetic dipole force and the magnetic levitational force during the self-assembly process in the inverse magnetic fluid, especially for the nonmagnetic microparticles with smaller diameter. The fluorescence area ratios of 3 *μ*m nonmagnetic microparticles are increased by employing the magnetic field induced self-assembly, with the average ratio of 112.92%. Moreover, the scattering of fluorescent nonmagnetic microparticle is absorbed by the surrounding ferrofluid, which can provide a quantitative result to retrospect the infection history. This study confirms that the inverse magnetic fluids have following advantages: low cost, no scattering effect, stable fluorescence intensity, and relatively low magnetic resistance. According to these advantages, a prototype design for the novel detection of COVID-19 based on the magnetic field induced self-assembly in the inverse magnetic fluids is proposed, which could support the epidemic prevention and control.

## Data Availability

The data that support the findings of this study are available from the corresponding author upon reasonable request.
